# Interspecific Competition Between *Eotetranychus sexmaculatus* Riley and *Oligonychus biharensis* Hirst (Acari: Tetranychidae)

**DOI:** 10.3390/insects16010096

**Published:** 2025-01-17

**Authors:** Lijiu Zheng, Yong Zhang, Xia Shi, Wei Gan, Fangping Zhang, Yueguan Fu, Ya Liu, Junyu Chen, Zhengpei Ye

**Affiliations:** 1 Environment and Plant Protection Institute, Chinese Academy of Tropical Agricultural Sciences, Haikou 571101, China; zhenglijiu@163.com (L.Z.);; 2 Hainan Provincial Engineering Research Center for the Breeding and Industrialization of Natural Enemies, Haikou 571101, China; 3 Honghe Tropical Agriculture Institute of Yunnan, Honghe 661300, China; 4 Sanya Research Academy, Chinese Academy of Tropical Agriculture Science, Sanya 572000, China

**Keywords:** spider mite, temperature, intraspecific competition, population dynamics, intrinsic rate of increase, interspecific competition coefficients

## Abstract

*Eotetranychus sexmaculatus* has long been recognized as an important spider mite pest of rubber trees. Recently, increasing damage from *Oligonychus biharensis* has elevated its importance as a key spider mite pest. These two species share highly overlapping ecological niches, with outbreaks strongly associated with high temperatures and drought stresses. However, little is known regarding the potential competition between these spider mite species and the role of temperature in shaping these interactions. This study investigates the development and reproduction of *E. sexmaculatus* and *O. biharensis* at varying population densities, and evaluates their dynamics at 27 °C, 30 °C, and 33 °C. Five initial population densities of *E. sexmaculatus* and *O. biharensis* were evaluated in mixed populations: 0:30, 10:20, 15:15, 20:10, and 30:0. The results demonstrate both intra- and interspecific competition between *E. sexmaculatus* and *O. biharensis*. At all three intraspecific densities, the survival rate and lifespan of both species declined as density increased, with fecundity also decreasing at higher densities. Single-species populations of each mite were larger in number when compared with mixed populations. Across all treatments, the mean and peak values of the intrinsic rate of increase (r_m_) were greater in *O. biharensis* than in *E. sexmaculatus*. Additionally, increasing temperatures accelerated the displacement of *E. sexmaculatus* by *O. biharensis*, regardless of the initial population density. When the mixed populations of *E. sexmaculatus* and *O. biharensis* were at densities of 10:20, the highest interspecific competition coefficients were obtained at 33 °C, with values of 0.6591. In conclusion, *O. biharensis* consistently outcompeted *E. sexmaculatus* at all tested temperatures, irrespective of initial densities, providing valuable insights into the competitive dynamics of dominant rubber tree pests.

## Simple Summary

*Eotetranychus sexmaculatus* and *Oligonychus biharensis* are devastating mite pests of the valuable tropical crop, rubber trees. Outbreaks of both spider mite species are associated with high temperatures and drought stresses. To evaluate both intra- and interspecific competition and the effects of temperature, we analyzed the population dynamics of *E. sexmaculatus* and *O. biharensis* under different temperature treatments. The results reveal the presence of both types of competition and demonstrate that *O. biharensis* is always more competitive than *E. sexmaculatus*, regardless of the temperature and the initial ratio. Therefore, *O. biharensis* has the potential to become a dominant pest of rubber trees. These findings provide a theoretical basis for early detection and effective management strategies for spider mites on rubber trees.

## 1. Introduction

**Figure 1:**
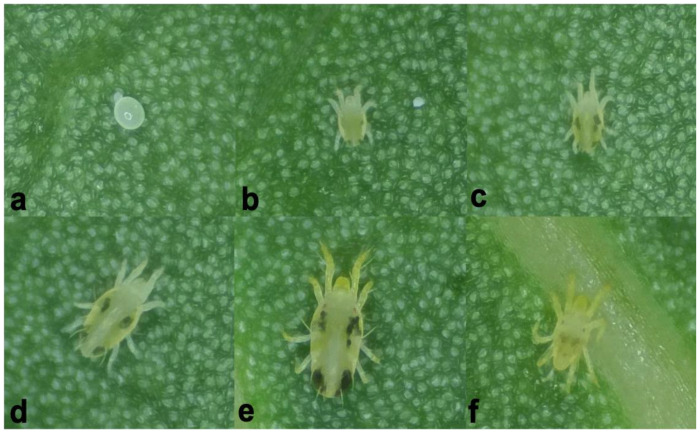
The developmental stages of *E. sexmaculatus*: (**a**) egg, (**b**) larva, (**c**) protonymph instar, (**d**) deutonymph instar, (**e**) adult female mite, (**f**) adult male mite.

**Figure 2:**
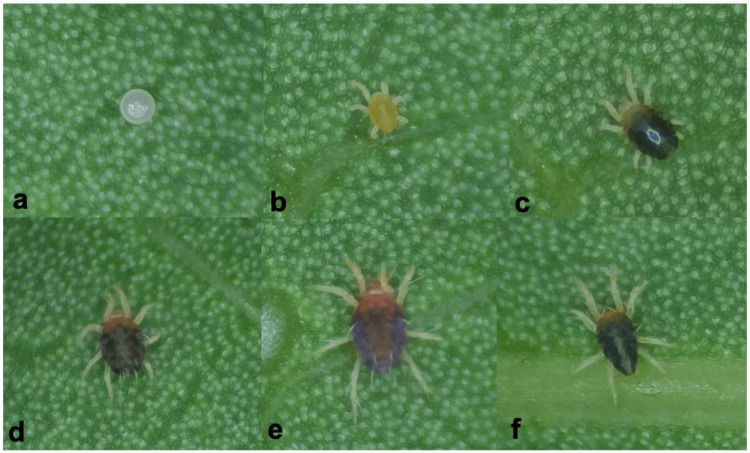
The developmental stages of *O. biharensis*: (**a**) egg, (**b**) larva, (**c**) protonymph instar, (**d**) deutonymph instar, (**e**) adult female mite, (**f**) adult male mite.

**Figure 3:**
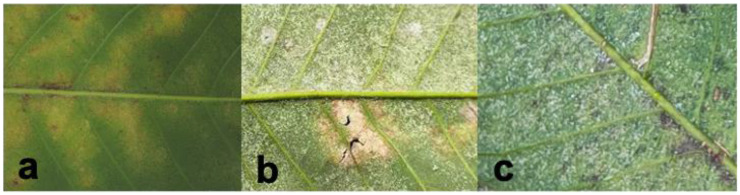
Field damage status of *E. sexmaculatus* and *O. biharensis*: (**a**) *E. sexmaculatus* damaged alone, (**b**) *O. biharensis* damaged alone, (**c**) combination of *E. sexmaculatus* and *O. biharensis* damaged.

Natural rubber is a highly important tropical crop and an essential industrial material [1,2]. Belonging to the mite family Tetranychidae, *Eotetranychus sexmaculatus* (Figure 1) is a widely distributed phytophagous mite that has constituted a critical pest problem for rubber tree (*Hevea brasiliensis*) plantations since the 1980s [1,3]. Our recent field survey of rubber trees revealed the presence of the spider mite *Oligonychus biharensis* (Figure 2), which belongs to the Tetranychidae family. Since 2016, *O. biharensis* has increasingly damaged rubber tree leaves, especially in high-cultivation regions such as Yunnan and Hainan. Unlike *E. sexmaculatus*, *O. biharensis* has a shorter history of rubber tree destruction and a limited geographic distribution. While these species may occur independently or simultaneously within a field (Figure 3), most severe rubber tree damage in China is attributed to *E. sexmaculatus*. Recently, reports of *O. biharensis* infestations have increased, indicating its rise as an important rubber tree pest [2]. *Eotetranychus sexmaculatus* and *O. biharensis* share a highly overlapping ecological niche, with both inhabiting and damaging the leaves and occasionally petioles of rubber trees. Moreover, both species are distributed across Hainan, Yunnan, and Guangdong and can be active in the spring, summer, and fall. This overlap likely results in interspecific competition between the two mites, which may be affected by environmental factors such as temperature. Interspecific competition occurs when different species at the same trophic level compete for the same resources to establish their populations [4,5,6]. Competitive interactions are generally asymmetric, leading to fluctuations in population sizes or even competitive displacement [7]. Therefore, further exploration is needed to determine if *O. biharensis* could replace *E. sexmaculatus* as the dominant rubber tree pest or whether they will form a stable coexistence.

Interspecific competition is influenced by various complex ecological factors, including temperature, host, and initial population size [5,8]. Intense interspecific competition has been documented within the same ecological niches in different temperature ranges [9]. For example, competition among the booklice species *Liposcelis bostrychophila*, *Liposcelis decolor*, and *Liposcelis paeta* has been reported at 25 °C and 30 °C [9]. As temperature adaptations vary across insect or mite species, environmental changes often have asymmetric effects on their competition. While the population dynamics of mites and insects are highly dependent on environmental temperature [10,11,12], they are also strongly influenced by the initial population size. For instance, when *L. bostrychophila* or *L. decolor* have larger populations, *L. bostrychophila* has a competitive advantage [9]. Competitive displacement is the most brutal result of interspecific competition and arises when two groups cannot coexist in the same environment. This phenomenon is related to ecological niche competition, host adaptability, and high-temperature adaptability [13]. For instance, the B biotype of *Bemisia tabaci* disrupts the mating of other biotypes, decreasing the proportion of females and contributing to population displacement.

Previous studies of *E. sexmaculatus* and *O. biharensis* have primarily focused on their biological characteristics, resistance mechanisms, and genetic functions [14,15,16,17]. However, their capacity for intra- and interspecific competition remains a mystery. Both *E. sexmaculatus* and *O. biharensis* are strongly adaptable on the rubber variety RRIM600. While *E. sexmaculatus* mainly damages the tender leaves of rubber plants [18], *O. biharensis* consumes rubber trees as well as longan and litchi [19]. The population adaptability of *E. sexmaculatus* and *O. biharensis* was assessed at 21–33 °C. At 27 °C, the developmental duration of *E. sexmaculatus* is shorter than *O. biharensis*, while the lifespan of female adult mites is longer than *O. biharensis*. The fecundity of spider mites is relatively high at 24–33 °C. Studies have demonstrated that a temperature of 27–30 °C benefits *E. sexmaculatus* population growth, whereas *O. biharensis* is more suited for growth at 33 °C [20]. The overlap in these habitats indicates the potential for competition, although it is unclear if this has led to a change in the pattern of mite pest occurrence. Additionally, the role of environmental effects like temperature on these changes is unknown. This study examines the dynamics of *E. sexmaculatus* and *O. biharensis* in both single and mixed populations in moderate (27 °C) and high (30 °C and 33 °C) temperatures. Moreover, we calculated the intrinsic increase rate of the two spider mites and their interspecific competition coefficients to clarify the extent of the competition. This study provides a theoretical basis for pest forecasting and the effective control of rubber tree pests. By understanding the occurrence of spider mites, early monitoring and timely prevention and control can be achieved to limit the loss of glue production.

## 2. Materials and Methods

### 2.1. Mite Collection and Rearing

*E. sexmaculatus* and *O. biharensis* populations were collected from the rubber plantation in the Chinese Academy of Tropical Agriculture experimental field in Danzhou, Hainan Province (longitude: 109°28′9′′ E, latitude: 19°32′2′′ N). The mites were continuously reared on the leaves of the rubber tree variety Reyan 73397 for several generations in a laboratory setting (27 ± 1 °C, 70 ± 5% RH, L:D = 14:10). All experimental mites were maintained on Reyan 73397 leaves. *E. sexmaculatus* and *O. biharensis* used during these experiments were offspring produced by parents for continuous breeding, without combining with wild-type individuals.

### 2.2. Investigations on the Damage Caused by Spider Mites on Rubber Trees

Four rubber plantations in Huanglian Mountain, Mengla County, Yunnan Province were randomly selected and surveyed for *E. sexmaculatus* and *O. biharensis* presence and damage using the inter-row continuous plant method (longitude and latitude: 101°41′4′′ E and 21°82′7′′ N, 101°41′5′′ E and 21°83′3′′ N, 101°41′6′′ E and 21°83′0′′ N, 101°41′7′′ E and 21°83′2′′ N. From each plantation, 20 branches were collected from individual trees, with five leaves per branch analyzed for damage caused by *E. sexmaculatus* and *O. biharensis*. Twenty trees were selected from each rubber plantation, representing a total of eighty trees. A total of 400 rubber tree leaves were assessed. Both single-species and mixed populations were quantified, and the rate of leaf injury was calculated. No pesticides were applied to the field prior to the survey.

### 2.3. Mite Development and Reproduction in Single and Mixed Populations

Single population: The eggs of *E. sexmaculatus* or *O. biharensis* were obtained from the laboratory colonies and placed on leaf discs in groups of 5, 10, and 20. Each population was monitored once every 24 h and all treatments were replicated six times. Leaf discs (1.5 × 1.5 cm) were replenished every three days, and all experiments were maintained in an artificial climate box (27 ± 1 °C, 75 ± 5% RH, L:D = 14:10). The development duration and survival rate of each mite species were recorded during the juvenile stage, while fecundity and lifespan were observed during the adult stage.

Mixed population: Interspecific competition responses were measured by placing the eggs of both *E. sexmaculatus* and *O. biharensis* on the same leaf discs. Treatments consisted of *E. sexmaculatus* and *O. biharensis* eggs ratios of 5:5, 5:15, 10:10, and 15:5. Populations were monitored every 24 h and replicated six times. Leaf discs were replenished every three days, and all experiments were maintained in an artificial climate box (27 ± 1 °C, 75% ± 5% RH, L:D = 14:10). The developmental duration and survival rate of each mite species were recorded during the juvenile stage, while fecundity and lifespan were documented during the adult stage.

### 2.4. Interspecific Competition Under Various Temperatures

To investigate the role of temperature on interspecific competition, *E. sexmaculatus* and *O. biharensis* were assessed at five initial densities (0:30, 10:20, 15:15, 20:10, and 30:0), with a female-to-male ratio of 1:1, in artificial climatic chambers set to 27 °C, 30 °C, and 33 °C (75 ± 5% RH, L:D = 14:10), 30 pairs of spider mites were utilized, totaling 60 individuals. Adult virgin male and female mites were selected and transferred onto whole rubber tree leaves (96 cm^2^), with each treatment replicated three times. Leaves were refreshed every three days. The number of eggs, larvae, nymphs, and adult females and males of each species were recorded every five days. Each mixed population experiment concluded when one species was eliminated. Single-population experiments were concluded after all mixed-population experiments were completed.

### 2.5. Data Analysis

Each species’ data were analyzed independently using a three-way ANOVA, with temperature, initial density, and rearing time as the primary effects, and population size as the response variable. All analyses were conducted using Excel (version 2010) and SPSS (version 26.0), employing a one-way ANOVA and Tukey’s multiple comparisons. Graphs were generated using Graphpad Prism (version 9.4). The results are presented as the mean ± standard error. The specific calculation formula for the relevant indicator parameters is as follows:

(1) *R* = $X_{i}$/*T* × 100%, where *R* is the rate of leaf injury, *X* represents the total number of leaves with mite hazards, *i* is (1) the total number of leaves damaged by *E. sexmaculatus* alone, (2) the total number of leaves damaged by *E. sexmaculatus* alone and in combination, (3) the total number of leaves damaged by *O. biharensis* alone, and (4) the total number of leaves damaged by *O. biharensis* alone and in combination, respectively. *T* denotes the total number of leaves assessed [21].
(2) $\bar{D}$ = $\sum (x_{i}\times n_{i}\;)/n$, where *D* represents the development duration, *x* is the number of developmental days, *i* represents egg, larvae, protonymph instar, deutonymph instar and egg–adult, respectively (the same below). Similarly, *n* denotes the total number of spider mites at different developmental stages. Generation time represents the total developmental time from egg to adult mite [5].
(3) $\bar{S}$ = *C_i_*/*N* × 100%, where *S* is the survival rate, *C* represents the number entering the next stage, and *N* denotes the number completing previous stage [5].
(4) $\bar{F}$ = $\sum (f_{i}/n_{i})$, where *F* represents fecundity (eggs/female), *f* represents the total number of eggs laid on that day, and *n* denotes the total number of adult female mites on that day. Finally, *i* represents days [5].
(5) $\bar{L}$ = ∑(d×n)/*N*, where *L* denotes lifespan, *d* is the survival days of female adult mites on that day, *n* represents the number of female adult mites, and *N* indicates the total number of female adult mites [5].
(6) *R*_0_ = *N_t_*/*N*_0_, *r_m_* = ln*R*_0_/*t*, where R_0_ represents the net increase rate, N_t_ represents population size at time *t*, and *N*_0_ indicates population size at the initial time, *r_m_* represents the intrinsic rate of increase, *t* represents the number of days of investigation time [22].
(7) *αij* = $\sum p_{i}p_{j}/\sqrt{\left(\sum p_{i}^{2}\right)(\sum p_{j}^{2}})$, where *αij* represents interspecific competition coefficient, *αij* denotes the competition coefficient of species *i* and *j* sharing the same resource, and *Pi* and *Pj* represent the proportions of species *i* and *j* in each resource sequence [23].

## 3. Results

### 3.1. Spider Mite Damage in Rubber Plantations

**Figure 4:**
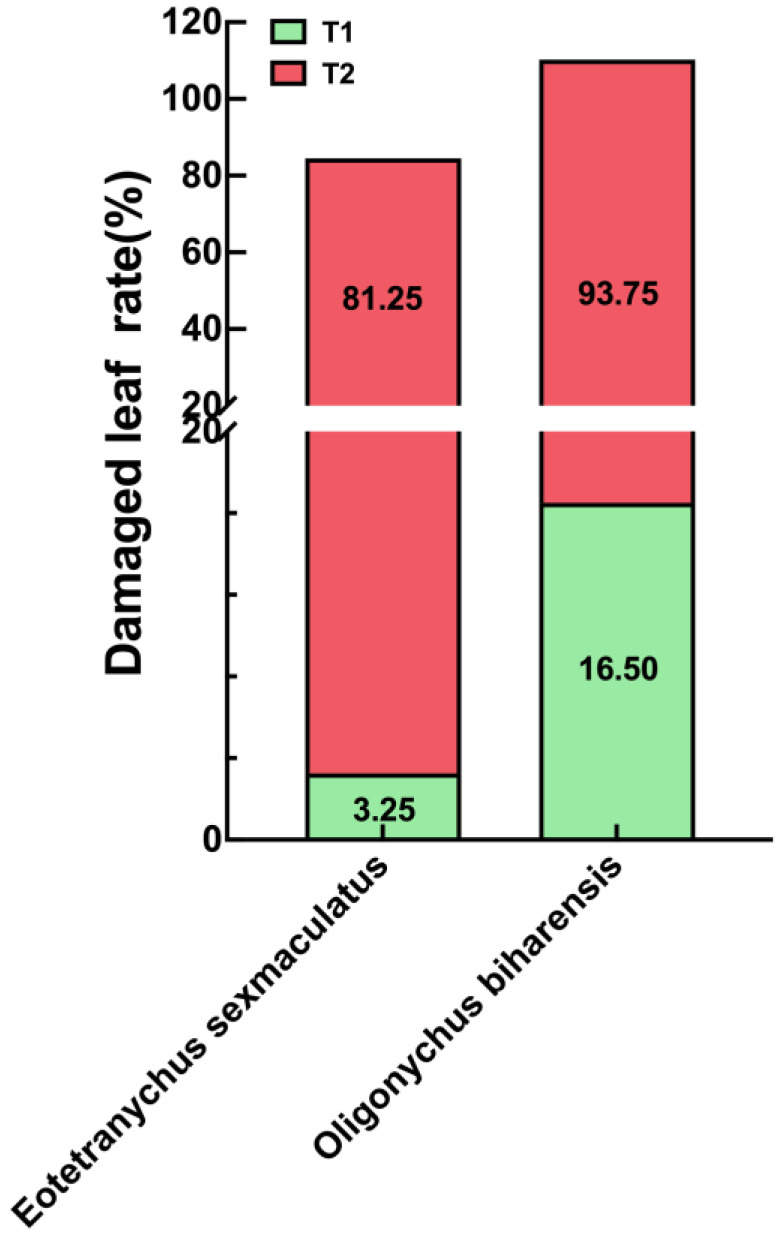
Damage caused by *E. sexmaculatus* and *O. biharensis* in rubber plantations. (**T1**) The damage rate of single species, (**T2**) overall damage rate, the total damage rate (including both single and mixed populations of two species). The values in the bars represent damaged leaf rate.

**Figure 5:**
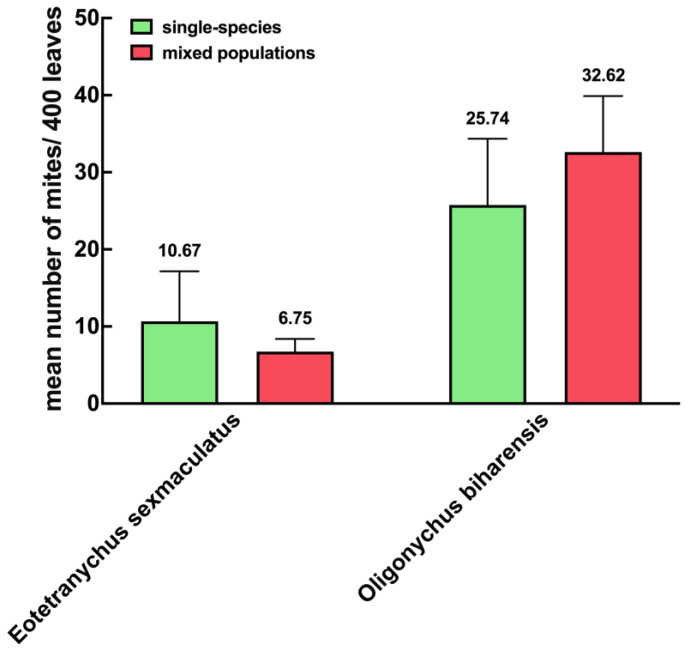
Population sizes of *E. sexmaculatus* and *O. biharensis* in rubber plantations. The values of figure are presented as mean ± standard error.

Our survey of rubber plantations revealed both individual and mixed populations of *E. sexmaculatus* and *O. biharensis*. When occurring independently, *E. sexmaculatus* populations averaged 10.67 individuals, causing a leaf damage rate of 3.25%, while *O. biharensis* populations averaged 25.74 individuals, with a leaf damage rate of 16.50%. When occurring in mixed populations, the average *E. sexmaculatus* population reduces to 6.75. In contrast, the population size of *O. biharensis* increased to 32.62. The leaf damage rate of *E. sexmaculatus* and *O. biharensis* increases to 81.25% and 93.75%. This result suggests that *O. biharensis* inflicts more significant damage to rubber trees than *E. sexmaculatus*. Moreover, the population of *O. biharensis* is 4.83 times greater than that of *E. sexmaculatus* in the mixed populations (Figure 4 and Figure 5).

### 3.2. Effects of Mixed Populations on Spider Mite Development

**Figure 6:**
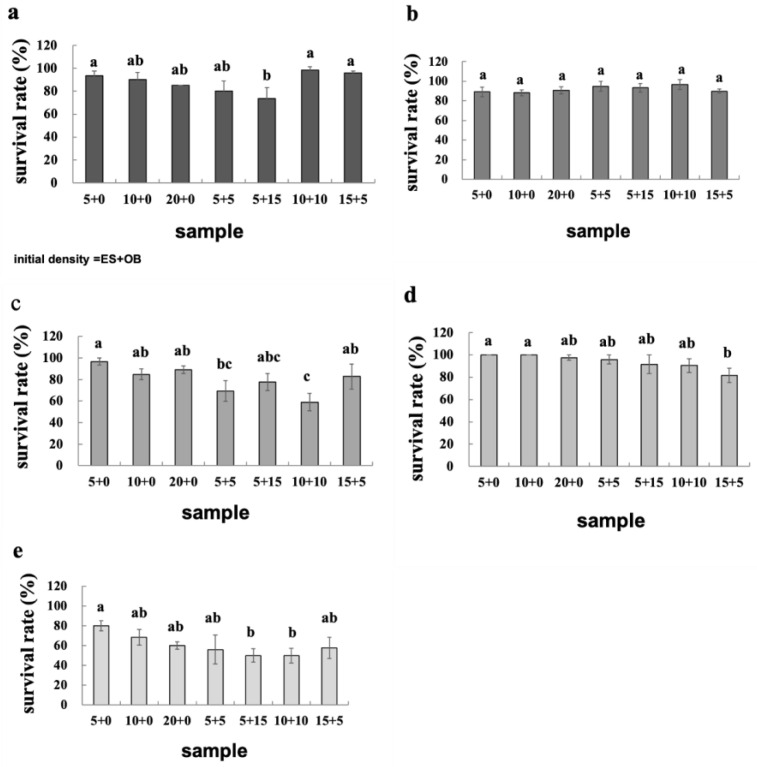
Survival rates of *E. sexmaculatus* at the (**a**) egg, (**b**) larvae, (**c**) protonymph instar, (**d**) deutonymph instar, (**e**) and egg—adult. (ES) *E. sexmaculatus*, (OB) *O. biharensis*. Bars represent mean ± standard error. Different letters above the error bars represents significantly different (one-way ANOVA, *p* < 0.05).

**Figure 7:**
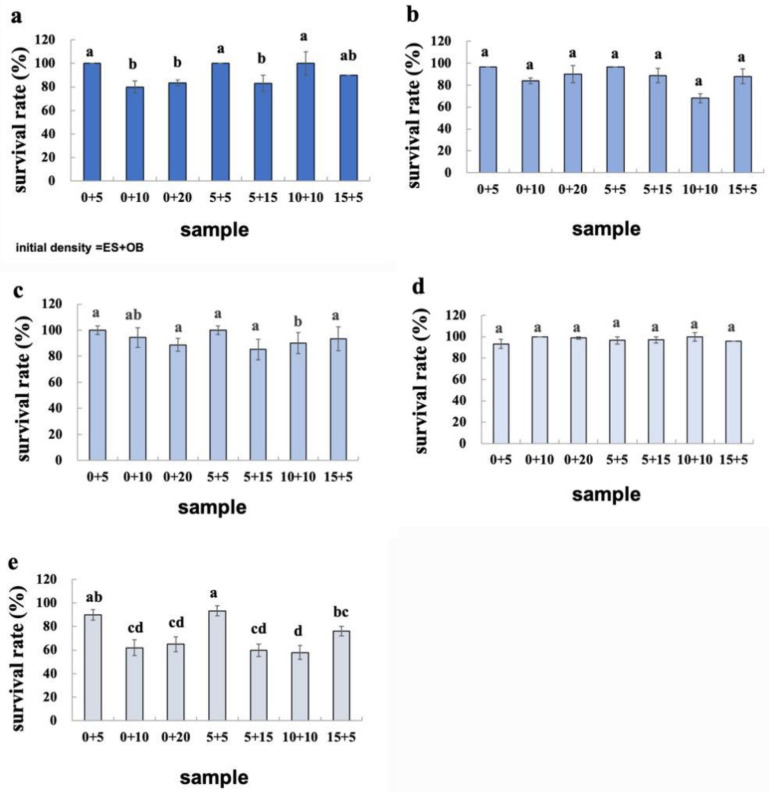
Survival rates of *O. biharensis* at the (**a**) egg, (**b**) larvae, (**c**) protonymph instar, (**d**) deutonymph instar, and (**e**) egg—adult. (ES) *E. sexmaculatus*, (OB) *O. biharensis*. Bars represent mean ± standard error. Different letters above the error bars represents significantly different (One-way ANOVA, *p* < 0.05).

Our study of density-dependent responses to intraspecific competition in *E. sexmaculatus* and *O. biharensis* revealed no significant difference in generation time across the three treatment densities (Table 1). The survival rates of both spider mite species declined progressively as intraspecific density increased. Both species showed the highest survival rate at a density of five mites per disc across all developmental stages. Additionally, the survival rate of both species from egg to adult decreased with increased density (Figure 6 and Figure 7). The highest-density group had a survival rate approximately 20% lower than that of the lowest-density group.

In mixed populations, both *E. sexmaculatus* and *O. biharensis* showed a significantly shorter development duration when in a 5:5 ratio, while no significant differences were observed in the remaining treatments. The developmental duration is influenced by density. Except for in the 5:5 treatment, the generation time of *E. sexmaculatus* was longer in mixed populations than in single ones, suggesting that this coexistence prolongs the species’ developmental period (Table 1).

No significant differences were observed in the survival rate of *E. sexmaculatus* from egg to adult in any of the four density treatments. The survival rate of *E. sexmaculatus* from egg to adult was reduced when the spider mites coexisted than when in isolation. The survival rate of *O. biharensis* from egg to adult was highest at an initial density of five mites per disc, whether in singular or mixed populations (Figure 6 and Figure 7).

### 3.3. Effects of Mixed Populations on Spider Mite Reproduction

**Figure 8:**
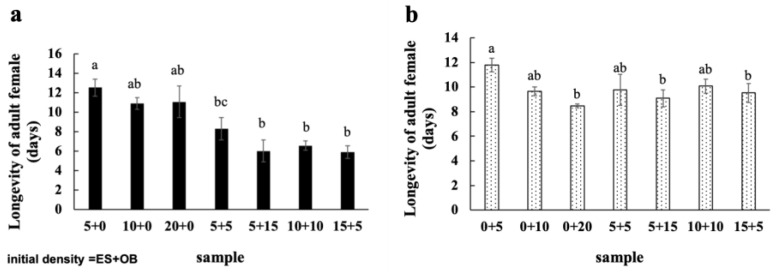
Lifespans of female adult (**a**) *E. sexmaculatus* and (**b**) *O. biharensis*. (ES) *E. sexmaculatus*, (OB) *O. biharensis*. Bars represent mean ± standard error. Different letters above the error bars represent significant differences (one-way ANOVA, *p* < 0.05).

**Figure 9:**
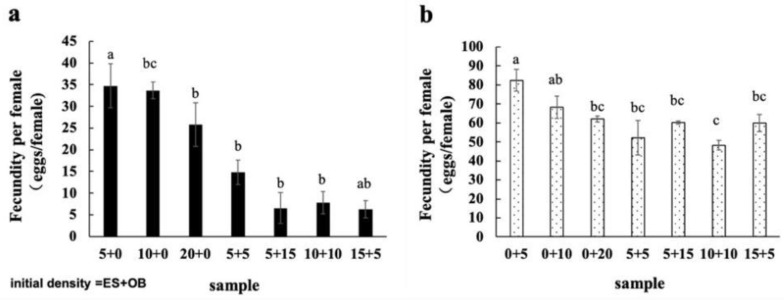
Fecundity of adult female (**a**) *E. sexmaculatus* and (**b**) *O. biharensis*. (ES) *E. sexmaculatus*, (OB) *O. biharensis*. Bars was presented mean ± standard error. Different letters above the error bars represents significant differences (one-way ANOVA, *p* < 0.05).

The longevity and fecundity of adult female mites in both species were lower across all mixing ratios compared to single-species populations, indicating that coexistence plays a significant role in population potential (Figure 8 and Figure 9). In single populations, the longevity of adult *E. sexmaculatus* (Figure 8a) and *O. biharensis* (Figure 8b) females decreased with increasing intraspecific density. The difference in the longevity of adult female *O. biharensis* mites was significant between the 0:5 and 15:5 groups.

### 3.4. Interspecific Competition Between Mite Species at Different Temperatures

The interactions between temperature and initial density, temperature and rearing time, and initial density and rearing time significantly affected the population size of *E. sexmaculatus*. In contrast, *O. biharensis* was strongly influenced only by the interactions between temperature and rearing time, as well as by initial density and rearing time (*p* < 0.05) (Table 2).

**Figure 10:**
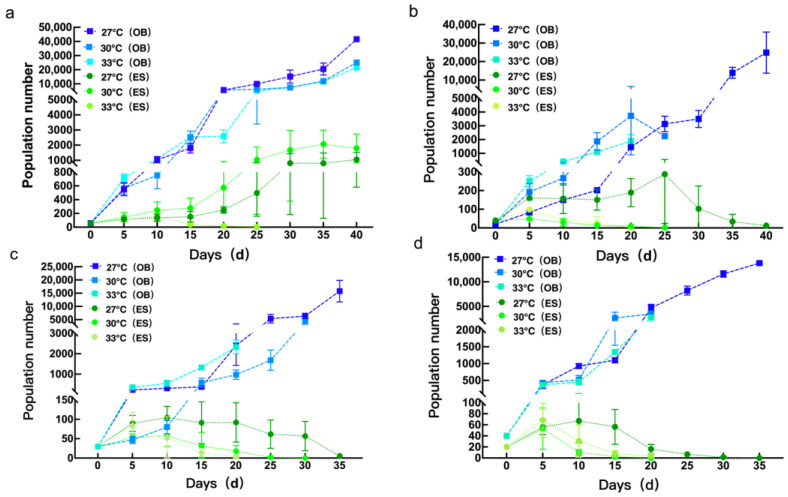
Effect of temperature on the population dynamics of (**a**) single-species populations and *E. sexmaculatus* to *O. biharensis* ratios of (**b**) 20:10, (**c**) 15:15, and (**d**) 10:20. (ES) *E. sexmaculatus*, (OB) *O. biharensis*.

In single-species treatments, the size of both spider mite populations was heavily dependent on temperature and differed significantly from the 5th to the 40th day (*p* < 0.05). The population sizes of *E. sexmaculatus* was largest at 30 °C compared to that at 27 °C and 33 °C at all observation time points. When the population sizes of *E. sexmaculatus* reached their peaks at 27 °C, 30 °C, and 33 °C, respectively, they increased to 17.52, 34.40, and 2.64 times the initial number, respectively. The population size of *O. biharensis* showed an overall increasing trend. From the 5th to the 15th day, the *O. biharensis* population size was greater at 33 °C compared to that at 27 °C and 30 °C. From the 20th to the 40th day, the *O. biharensis* population size was greater at 27 °C compared to at 30 °C and 33 °C. At the end of the observation, the population sizes of *O. biharensis* at 27 °C, 30 °C, and 33 °C, increased to 693.32, 417.94, and 357.76 times the initial number, respectively (Figure 10a).

When mixed populations initially comprised 20 *E. sexmaculatus* and 10 *O. biharensis* mites, each species’ population size changed drastically from the 5th to the 40th day (*p* < 0.05). At 27 °C, *E. sexmaculatus* dominated until the 10th day, when *O. biharensis* surpassed it in terms of population size. At 27 °C, *E. sexmaculatus* briefly dominated during the first 10 days when containing initially higher populations. At 27 °C, 30 °C, and 33 °C, when the population sizes of *E. sexmaculatus* reached their peak, they increased to 7.19, 1.21, and 2.47 times the initial number, respectively. When the population sizes of *O. biharensis* reached their peak, they increased to 1241.58, 186.37, and 94.73 times their initial number at these three temperatures, respectively (Figure 10b).

Mixed populations beginning with 15 *E. sexmaculatus* and 15 *O. biharensis* mites showed significant population fluctuations from the 5th to the 35th day (*p* < 0.05). The population of *O. biharensis* dominated throughout the entire observation period. At 27 °C, 30 °C, and 33 °C, when the population sizes of *O. biharensis* reached their peaks, they increased to 526.22, 139.34, and 77.73 times the initial number, respectively. For *E. sexmaculatus*, their peak population sizes increased to 7.61, 1.90, and 2.78 times the initial number at three tested temperatures, respectively. The population of *O. biharensis* generally increased, whereas the population of *E. sexmaculatus* initially increased and subsequently declined (Figure 10c).

When beginning with 10 *E. sexmaculatus* and 20 *O. biharensis* mites, the population sizes of both mites differed significantly from the 5th to the 35th day (*p* < 0.05). The population of *O. biharensis* dominated throughout this entire period. At 27 °C, 30 °C, and 33 °C, when the population sizes of *O. biharensis* reached peaks, they increased to 345.24, 86.34, and 67.88 times the initial number, respectively. This was much larger than the population sizes observed in *E. sexmaculatus*, their peak population sizes increased to 3.35, 2.65, and 3.42 times the initial number, respectively (Figure 10d).

Across all temperatures, the population size of each species in a combined population is lower than in isolation. When cohabitating, *O. biharensis* populations grew much larger than *E. sexmaculatus*, eventually replacing it in all treatments. This phenomenon was intensified by higher temperatures, which shortened the coexistence duration. For example, *E. sexmaculatus* was replaced by *O. biharensis* as early as the 20th day at 33 °C (Figure 10).

### 3.5. Intrinsic Rate of Increase Under Different Temperatures

**Figure 11:**
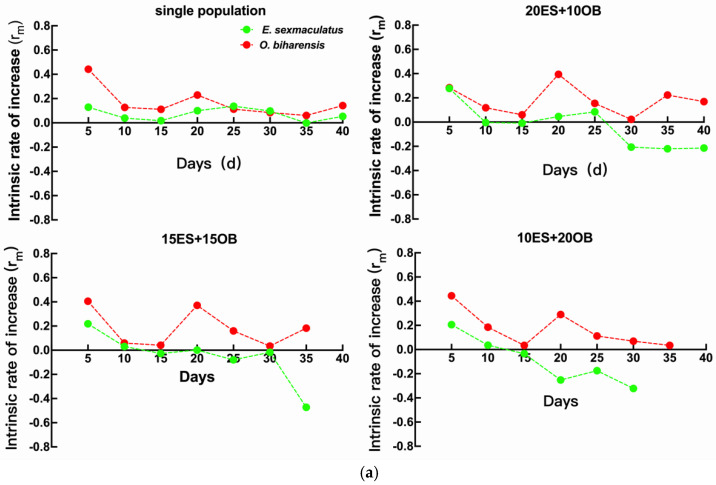

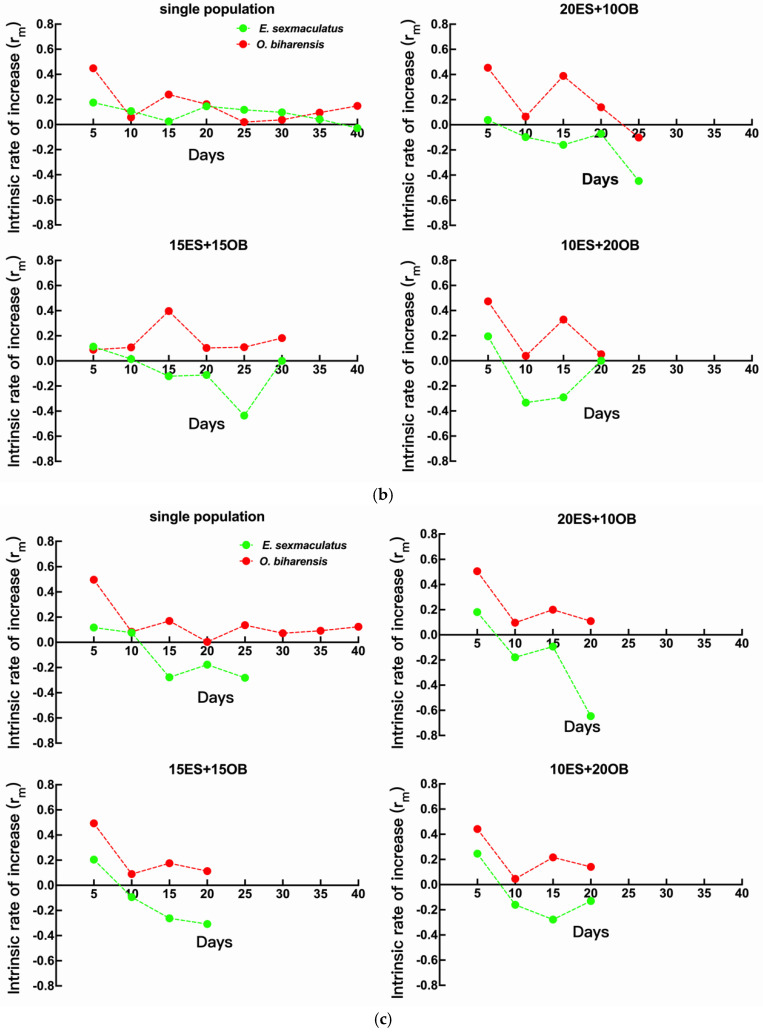
The intrinsic rate of increase in *E. sexmaculatus* and *O. biharensis* populations at the same temperature. (**a**) Effect of 27 °C temperatures on the r_m_ of (ES) *E. sexmaculatus* and (OB) *O. biharensis*. (**b**) Effect of 30 °C temperatures on the r_m_ of (ES) *E. sexmaculatus* and (OB) *O. biharensis*. (**c**) Effect of 33 °C temperatures on the r_m_ of (ES) *E. sexmaculatus* and (OB) *O. biharensis*.

In single-species populations, the highest r_m_ was observed at 30 °C for *E. sexmaculatus* and at 33 °C for *O. biharensis*. For both species, r_m_ values peaked on the 5th day at all tested temperatures, except for *E. sexmaculatus* at 27 °C, which peaked on the 25th day. The r_m_ was greater in *O. biharensis* than in *E. sexmaculatus*, except on the 25th and 30th days under 27 °C and 30 °C (Figure 11).

Mixed populations initiating with 20 *E. sexmaculatus* and 10 *O. biharensis* mites contained the largest r_m_ values at 27 °C in *E. sexmaculatus* and 33 °C in *O. biharensis*. For both species, the largest r_m_ values were recorded on the 5th day, except for *O. biharensis* at 27 °C which peaked on the 20th day. The r_m_ values of *O. biharensis* were greater than those of *E. sexmaculatus* across all treatments (Figure 11).

When mixed populations started with 15 *E. sexmaculatus* and 15 *O. biharensis*, the largest r_m_ values were documented at 27 °C in *E. sexmaculatus* and 33 °C in *O. biharensis*. For both species, the largest r_m_ values were obtained on the 5th day, except for *O. biharensis* at 30 °C, which was the highest on the 15th day. Overall, *O. biharensis* consistently exhibited greater r_m_ values than *E. sexmaculatus*, except on the 5th day at 30 °C (Figure 11).

Mixed populations containing 10 *E. sexmaculatus* and 20 *O. biharensis* mites reported the largest r_m_ values at 33 °C in *E. sexmaculatus* and 30 °C in *O. biharensis*, both peaking on the 5th day. The r_m_ of *O. biharensis* was consistently greater than *E. sexmaculatus* across all temperatures (Figure 11).

In single-species populations at 27 °C and 30 °C, the r_m_ of *E. sexmaculatus* was greater than that of mixed populations, except on the 5th day. At 33 °C, the r_m_ of *E. sexmaculatus* in both single and mixed populations exhibited a decreasing trend after the 10th day, suggesting that high temperatures negatively impact population growth and intensify interspecific competition. Except on the 5th day at 30 °C, the r_m_ values of *O. biharensis* were greater than *E. sexmaculatus* across all temperatures and population densities, demonstrating the highly competitive potential of *O. biharensis* (Figure 11).

### 3.6. Interspecific Competition Coefficients Under Different Temperatures

When the initial population of *E. sexmaculatus* was dominant, the interspecific competition coefficient at 30 °C was greater than that at 27 °C and 33 °C, but the differences among the three were not significant. When two species initially coexisted in equal numbers, the interspecific competition coefficient at 27 °C was greater than that at 30 °C and 33 °C, and the difference between 27 °C and 30 °C was significant. When the initial number of *O. biharensis* dominated, the interspecific competition coefficient at 33 °C was the greatest, and there were significant differences from 33 °C to other two temperatures (Table 3).

## 4. Discussion

This study examined the individual and mixed occurrence of two spider mite species under field conditions and assessed their development and reproduction under different densities. We also assessed both intra- and interspecific competition between two rubber tree spider mites, *E. sexmaculatus* and *O. biharensis*, which share the same ecological niche. We analyzed the populations of these species in both single and mixed populations under different initial ratios and temperature conditions.

Field investigation demonstrated that two spider mite species damaged rubber plantations alone and in combination. Most of the leaves in rubber plantations were eaten and damaged by both spider mite species, and the damage caused by mixed populations was relatively high. The population of *O. biharensis* was significantly higher than *E. sexmaculatus*. In practical production, monitoring both spider mite species and implementing timely prevention and control measures are of critical importance.

Findings showed that the coexistence of two spider mite species prolonged the developmental period of *E. sexmaculatus*, limited its survival rate and fecundity, and reduced the fecundity of *O. biharensis*. The survival and reproduction of *O. biharensis* are greatly affected by its own density. These results support the understanding of competition between the two spider mite species.

Our results demonstrate that the total population size of cohabitating spider mite species was smaller than that of single-species populations at all tested temperatures. There is a known suppressive effect on the population growth of species competing for resources in the same ecological niche [24]. A previous study of *Tetranychus urticae*, *Panonychus ulmi*, and *Tetranychus viennensis* revealed the dominance of *Tetranychus urticae*, with *Tetranychus viennensis* prevailing in its absence, providing evidence for interspecific competition between spider mite species [25]. Dominant species can use ecological niche factors to displace weaker ones, as documented in whiteflies, thrips, and spider mites [26].

Our study found that interspecific competition was particularly intense at higher temperatures, with competition coefficients peaking at 33 °C. This is concordant with previous studies that similarly correlate interspecific competition with temperature. One such study on *Chilo partellus*, *Busseola fusca*, and *Sesamia calamistis* determined that the three generally coexist at 15 °C, 20 °C, 25 °C, and 30 °C; however, high temperatures favoured the survival of *C. partellus* larvae, leading to its dominance in warmer environments [24]. Such discoveries reinforce the trend that competing species with strong temperature tolerances often gain a competitive advantage under high- or low-temperature adversity. Our study showed that at 27 °C, *O. biharensis* contained a much larger population than *E. sexmaculatus* in both single and mixed populations, cementing its competitive advantage. We can infer that the interspecific competition between the two spider mite species alters the occurrence pattern of phytophagous mites on rubber trees, a process likely accelerated by high temperatures. Our findings demonstrate that the replacement of *E. sexmaculatus* by *O. biharensis* can be hastened by elevated temperatures. This behaviour is mirrored in other insect species. For instance, *L. bostrychophila* inhibited the population size of *L. decolor* and *L. paeta* at 25 °C and 30 °C [12]. At 30 °C, the juvenile survival rate of *C. maculatus* was higher than that of *C. chinensis*, while the reproductive capacities of both were greatly diminished [27]. Finally, *Sitophilus zeamais* and *Prostephanus truncatus* are known to coexist at 25–35 °C, with *S. zeamais* dominating at 25–30 °C, while *P. runcates* prevails at 35 °C [28].

In addition to temperature, the result of interspecific competition is influenced by the initial population density [29]. Species with higher preliminary densities may dominate in the initial competition; however, the species with a stronger competitive ability will gradually claim dominance. In this study, despite starting with a higher initial density, at 27 °C, *E. sexmaculatus* surrendered its dominance to *O. biharensis* after the 10th day. A certain population size or density accelerates interspecific competition among insects, potentially inhibiting population growth in one or both species [30]. A previous study revealed that the coexistence between *L. bostrychophila* and *L. decolor* led to decreases in population sizes for both, with reductions distinct from single-species populations [12].

The outcome of competition and the growth of phytophagous insect populations are known to be influenced by the intrinsic rate of increase [31]. The population growth potential of *E. sexmaculatus* and *O. biharensis* on the rubber variety RRIM600 was assessed, revealing large r_m_ for both species. Previous studies have reported the strong population growth ability of *E. sexmaculatus* on tender rubber leaves, with a r_m_ value of 0.1041 [18]. Our findings show that *O. biharensis* exhibits an even larger r_m_ value, reaching 0.457 on RRIM600. Therefore, *O. biharensis* has a stronger population growth potential than *E. sexmaculatus*. Interspecific competition affects the intrinsic rate of increase for both competing species, with the degree of influence determined by the strength of competitive ability. In our study, the mean r_m_ values in *O. biharensis* were greater than those of *E. sexmaculatus* from the 5th to the 40th day in both single and mixed populations, indicating a stronger competitive edge. This is supported by a previous study showing that the mean r_m_ values in *Tetranychus urticae* were greater than those of *Tetranychus truncatus* in both individual and mixed populations, indicating the strong competitiveness of *T. urticae* [30]. Additionally, the highly competitive advantage of *Blattella germanica* is reflected by its greater reproductive capacity and intrinsic rate of increase compared to *Symploce pallens* [32].

This study’s results indicate the occurrence and distribution of two spider mite species in the field, as well as inter-species competition, providing a foundation for monitoring the occurrence of two spider mite species. By understanding the occurrence of spider mites, early monitoring and timely prevention and control can be attained to limit the loss of glue production. When the field temperature reaches 30 °C, the monitoring of spider mite species should be strengthened.

## 5. Conclusions

Taken together, these results indicate interspecific competition between *E. sexmaculatus* and *O. biharensis*, two dominant pests of rubber trees. Our results demonstrate that *O. biharensis* contains a higher competitive potential than *E. sexmaculatus* at 27–33 °C. However, these studies were conducted in a laboratory setting, which may not be reflective of field conditions due to the influence of natural enemies, hosts, meteorological factors, and pesticides. Future studies should further investigate these interactions in field conditions, with a focus on the influence of natural enemies and the mechanisms underlying competitive displacement.

## Figures and Tables

**Table 1:** Development duration (days ± S.E.) of *E. sexmaculatus* and *O. biharensis* on different initial density.

Species	Initial Density	Egg	Larva	Protonymph Instar	Deutonymph Instar	Generation Time
*E. sexmaculatus*	0 + 5	4.93 ± 0.16 a	2.28 ± 0.23 a	1.73 ± 0.14 cd	1.57 ± 0.19 abc	10.49 ± 0.11 b
	0 + 10	4.00 ± 0.00 b	2.00 ± 0.00 a	2.82 ± 0.09 ab	1.10 ± 0.19 c	9.92 ± 0.18 bc
	0 + 20	4.13 ± 0.08 b	2.37 ± 0.13 a	1.22 ± 0.17 d	2.29 ± 0.33 a	9.99 ± 0.31 bc
	5 + 5	4.36 ± 0.18 b	2.20 ± 0.33 a	1.67 ± 0.26 cd	1.20 ± 0.20 bc	9.50 ± 0.22 c
	5 + 15	4.33 ± 0.21 b	2.53 ± 0.18 a	2.93 ± 0.21 ab	2.21 ± 0.36 ab	12.00 ± 0.45 a
	10 + 10	5.41 ± 0.19 a	2.24 ± 0.15 a	2.39 ± 0.20 bc	2.62 ± 0.37 a	12.67 ± 0.33 a
	15 + 5	4.43 ± 0.24 b	2.48 ± 0.45 a	3.33 ± 0.58 a	2.60 ± 0.56 a	12.84 ± 0.31 a
*F*, *df*1, *df*2, *p* value		8.308, 6, 35, *p* < 0.001	0.532, 6, 35, *p* < 0.001	7.728, 6, 34, *p* < 0.001	3.414, 6, 34, 0.015	22.269, 6, 34, 0.432
*O. biharensis*	0 + 5	4.63 ± 0.20 bc	1.97 ± 0.22 ab	1.68 ± 0.23 c	2.99 ± 0.53 a	11.27 ± 0.56 ab
	0 + 10	5.00 ± 0.03 ab	2.11 ± 0.12 ab	2.41 ± 0.37 bc	1.39 ± 0.25 b	10.94 ± 0.06 abc
	0 + 20	4.95 ± 0.02 ab	1.26 ± 0.13 c	2.50 ± 0.32 bc	1.68 ± 0.23 b	10.38 ± 0.20 bc
	5 + 5	4.10 ± 0.28 c	1.60 ± 0.13 bc	2.04 ± 0.38 c	1.83 ± 0.22 b	9.57 ± 0.28 c
	5 + 15	5.38 ± 0.16 a	1.74 ± 0.13 bc	3.64 ± 0.25 a	1.17 ± 0.17 b	11.93 ± 0.05 a
	10 + 10	5.43 ± 0.29 a	1.98 ± 0.26 ab	3.96 ± 0.41 a	1.65 ± 0.37 b	12.08 ± 0.91 a
	15 + 5	4.48 ± 0.23 bc	2.33 ± 0.21 a	3.39 ± 0.49 ab	2.05 ± 0.51 ab	12.21 ± 0.22 a
*F*, *df*1, *df*2, *p* value		5.689, 6, 35, *p* < 0.001	3.949, 6, 35, 0.004	6.113, 6, 32, *p* < 0.001	3.041, 6, 32, 0.018	4.688, 6, 33, 0.001

The data represents mean ± standard error. The means within the same column followed by different letters are significantly different (one-way ANOVA, *p* < 0.05). initial density = *E. sexmaculatus*+ *O. biharensis*.

**Table 2:** ANOVA parameters for the primary effects and their interactions.

Spider Mite	Factor	*df*	*F*	*p*
*E. sexmaculatus*	Temperature	2	5.100	0.007
	Initial density	3	40.163	*p* < 0.001
	Rearing time	7	3.679	0.001
	Temperature and initial density	6	3.032	0.008
	Temperature and rearing time	9	2.126	0.031
	Initial density and rearing time	18	4.547	*p* < 0.001
	Temperature and initial density and rearing time	21	0.579	0.927
*O. biharensis*	Temperature	2	7.308	0.001
	Initial density	3	45.195	*p* < 0.001
	Rearing time	7	271.637	*p* < 0.001
	Temperature and initial density	6	0.927	0.477
	Temperature and rearing time	9	3.269	0.001
	Initial density and rearing time	18	7.011	*p* < 0.001
	Temperature and initial density and rearing time	21	0.721	0.806

**Table 3:** Interspecific competition coefficients at different temperatures.

Temperature		Mixed Population	
	20 ES vs. 10 OB	15 ES vs. 15 OB	10 ES vs. 20 OB
27 °C	0.4157 ± 0.04 a	0.6466 ± 0.07 a	0.5388 ± 0.42 b
30 °C	0.5152 ± 0.06 a	0.3927 ± 0.04 b	0.5484 ± 0.16 b
33 °C	0.4976 ± 0.05 a	0.5441 ± 0.05 ab	0.6591 ± 0.05 a
*F*, *df*1, *df*2, *p*	6.493, 2, 6, 0.032	5.975, 2, 6, 0.037	1.017, 2, 6, 0.417

The data represents mean ± standard error. The means within the same column followed by different letters are significantly different (One-way ANOVA, *p* < 0.05). ES (*E. sexmaculatus*), OB (*O. biharensis*).

## Data Availability

The data presented in this study are available upon request from the corresponding author. The data are not publicly available.

## References

[1] Hao H.H., Li P.Z., Xu T.W., Wu Q.Q., Zhang F.P., Peng Z.Q. (2021). Preliminary evaluation of the control effect of two predatory mite species on *Eotetranychus sexmaculatus* in rubber trees in Hainan Province, China. Syst. Appl. Acarol..

[2] Liu Y., Nie Y., Chen J.Y., Lu T.F., Niu L.M., Jia J.J., Ye Z.P., Fu Y.G. (2022). Genetic diversity of three major spider mites damaging rubber trees. Syst. Appl. Acarol..

[3] Liang X., Chen Q., Wu C.L., Liu Y., Fang Y.J. (2020). Reference gene validation in *Eotetranychus sexmaculatus* (Acari: Tetranychidae) feeding on mite-susceptible and mite-resistant rubber tree germplasms. Exp. Appl. Acarol..

[4] Carbonell J.A., Céspedes V., Coccia C., Green A.J. (2020). An experimental test of interspecific competition between the alien boatman *Trichocorixa verticalis* and the native corixid *Sigara lateralis* (Hemiptera, Corixidae). Aquat. Invasions.

[5] Zhang G.F., Lovei G.L., Hu M., Wan F.H. (2014). Asymmetric consequences of host plant occupation on the competition between the whiteflies *Bemisia tabaci* cryptic species MEAM1 and *Trialeurodes vaporariorum* (Hemiptera: Aleyrodidae). Pest Manag. Sci..

[6] Li J.L., Liu S., Guo K., Zhang F., Qiao H.L., Chen J.M., Yang M.K., Zhu X., Xu R., Xu C.Q. (2018). Plant-mediated competition facilitates a phoretic association between a gall mite and a psyllid vector. Exp. Appl. Acarol..

[7] Denno R.F., McClure M.S., Ott J.R. (1995). Interspecific interactions in phytophagous insects: Competition reexamined and resurrected. Annu. Rev. Entomol..

[8] Mortazavi N., Fathipour Y., Talebi A.A. (2017). Interactions between two-spotted spider mite, *Tetranychus urticae* and greenhouse whitefly, *Trialeurodes vaporariorum* on strawberry. Syst. Appl. Acarol..

[9] Athanassiou C.G., Kavallieratos N.G., Throne J.E., Nakas C.T. (2014). Competition among Species of Stored-Product Psocids (Psocoptera) in Stored Grain. PLoS ONE.

[10] Lin M.Y., Lin C.H., Lin Y.P., Tseng C.T. (2020). Temperature-dependent life history of *Eutetranychus africanus* (Acari: Tetranychidae) on papaya. Syst. Appl. Acarol..

[11] Araujo F.G.D., Lima E.L.D., Costa E., Daud R.D. (2022). Influence of natural vegetation conservation on the distribution of mites in rubber tree crops. Syst. Appl. Acarol..

[12] Ferraz J.C.B., Neto A.V.G., Frana S.M.D., Silva P.R.R., Lima D.B.D. (2021). Temperature-dependent development and reproduction of *Oligonychus punicae* (Acari: Tetranychidae) on Eucalyptus. Syst. Appl. Acarol..

[13] Liu S.S., Barro P.J.D., Xu J., Luan J.B., Zang L.S., Ruan Y.M., Wan F.H. (2007). Asymmetric Mating Interactions Drive Widespread Invasion and Displacement in a Whitefly. Science.

[14] Chen W.M., Fu Y.G., Zhang F.P., Peng Z.Q. (2005). Effect of different varieties of litchi on the development and reproduction of *Oligonychus biharensis* (Hirst). Syst. Appl. Acarol..

[15] Kaimal S.G., Sheeja U.M., Ramani N. (2011). Ultrastructural elucidation of leaf damage on cassava induced by *Oligonychus biharensis* (Hirst) (Acari: Tetranychidae). Int. J. Acarol..

[16] Ji J., Zhang Y.X., Chen X., Lin J.Z. (2008). Responses to stimuli from *Oligonychus biharensis* Hirst (Acari: Tetranychidae) on loquat leaves by *Neoseiulus cucumeris* (Oudemans) (Acari: Phytoseiidae). Int. J. Acarol..

[17] Lu F.P., Chen Z.S., Lu H., Liang X., Zhang H.Y., Li Q., Chen Q., Huang H.S., Hua Y.W., Tian W.M. (2016). Effects of resistant and susceptible rubber germplasms on development, reproduction and protective enzyme activities of *Eotetranychus sexmaculatus* (Acari: Tetranychidae). Exp. Appl. Acarol..

[18] Zhang F.P., Zhu J.H., Li L., Han D.Y., Niu L.M., Chen J.Y., Fu Y.G. (2016). Effect of Rubber Leaves on the Selectivity and Growth Stages of *Eotetranychus sexmaculatus* at Different Growing Period. Chin. J. Trop. Crops.

[19] Zhang F.P., Fu Y.G., Jin Q.A., Zhang J.B. (2007). Development and fecundity of *Oligonychus biharensis* on three southern fruit crops. J. Fruit Sci..

[20] Wang W.H., Wan S.L., Zheng L.J., Zhang F.P., Chen J.Y. (2024). Evaluation of the temperature adaptation of three rubber tree pest mites based on their two-sex life table. Insects.

[21] Zhang T.F., Zhao T.P., Li X.D., Rao Y.F., Yang J.B., Yang Z.J. (2023). Study on the field control effect of *Arma chinensis* (Fallou) on *Spodoptera frugiperda* (J. E. Smith). Yunnan Agric..

[22] Li Y., Wei X., Zhang J., Xiang L., Feng H.Z. (2015). Interspecific competition between *Tetranychus urticae* and *Tetranychus truncates* on cotton. Acta Agric. Bor.-Oci. Sin..

[23] May R.M. (1975). Some notes on estimating the competition matrix. Ecology.

[24] Ntiri E.S., Calatayud P.A., Berg J.V.D., Schulthess F., Ru B.P.L. (2016). Influence of Temperature on Intra- and Interspecific Resource Utilization within a Community of *Lepidopteran Maize* Stemborers. PLoS ONE.

[25] Yan W.T., Qiu G.S., Zhou Y.S., Zhang H.J., Zhang P., Liu C.L., Zheng Y.C. (2010). Interspecific domino effects of three major pernicious mites in apple orchard. J. Fruit Sci..

[26] Zhang Q.C., Yan W.J., Wang J.G. (2022). Laboratory Assays of Density-Dependent Interspecific and Intraspecific Competition between *Aphis gossypii* and *Acyrthosiphon gossypii* (Hemiptera: Aphididae). J. Entomol. Sci..

[27] Kishi S., Nishida T., Tsubaki Y. (2009). Reproductive interference determines persistence and exclusion in species interactions. J. Anim. Ecol..

[28] Quellhorst H., Athanassiou C.G., Bruce A., Scully E.D., Morrison W.R. (2020). Temperature-Mediated Competition Between the Invasive *Larger Grain* Borer (Coleoptera: Bostrichidae) and the *Cosmopolitan Maize* Weevil (Coleoptera: Curculionidae). Environ. Entomol..

[29] Pascual S., Callejas C. (2004). Intra-and interspecific competition between biotypes B and Q of *Bemisia tabaci* (Hemiptera: Aleyrodidae) from Spain. Bull. Entomol. Res..

[30] Gamelon M., Vriend S.J.G., Engen S., Adriaensen F., Dhondt A.A., Evans S.R., Matthysen E., Sheldon B.C., Saether B.E. (2019). Accounting for interspecific competition and age structure in demographic analyses of density dependence improves predictions of fluctuations in population size. Ecol. Lett..

[31] Kim T.N., Underwood N., Inouye B.D. (2013). Insect herbivores change the outcome of plant competition through both inter-and intraspecific processes. Ecology.

[32] Bujang N.S., Lee C.Y. (2010). Interspecific competition between the Smooth cockroach *Symploce pallens* and the German cockroach *Blattella germanica* (Dictyoptera: Blattellidae) under different food and water regimes. Trop. Biomed..

